# A Novel Method of Aircraft Detection under Complex Background Based on Circular Intensity Filter and Rotation Invariant Feature

**DOI:** 10.3390/s22010319

**Published:** 2022-01-01

**Authors:** Xin Chen, Jinghong Liu, Fang Xu, Zhihua Xie, Yujia Zuo, Lihua Cao

**Affiliations:** 1 Changchun Institute of Optics, Fine Mechanics and Physics, Chinese Academy of Sciences, Changchun 130033, China; chenxin176@mails.ucas.ac.cn (X.C.); xufang@ciomp.ac.cn (F.X.); xiezhihua17@mails.ucas.ac.cn (Z.X.); zuoyujia@ciomp.ac.cn (Y.Z.); caolh@ciomp.ac.cn (L.C.); 2 University of Chinese Academy of Sciences, Beijing 100049, China

**Keywords:** remote sensing images, aircraft target detection, circular intensity filter, rotation invariant feature, vector of locally aggregated descriptors (VLAD)

## Abstract

Aircraft detection in remote sensing images (RSIs) has drawn widespread attention in recent years, which has been widely used in the military and civilian fields. While the complex background, variations of aircraft pose and size bring great difficulties to the effective detection. In this paper, we propose a novel aircraft target detection scheme based on small training samples. The scheme is coarse-to-fine, which consists of two main stages: region proposal and target identification. First, in the region proposal stage, a circular intensity filter, which is designed based on the characteristics of the aircraft target, can quickly locate the centers of multi-scale suspicious aircraft targets in the RSIs pyramid. Then the target regions can be extracted by adding bounding boxes. This step can get high-quality but few candidate regions. Second, in the stage of target identification, we proposed a novel rotation-invariant feature, which combines rotation-invariant histogram of oriented gradient and vector of locally aggregated descriptors (VLAD). The feature can characterize the aircraft target well by avoiding the impact of its rotation and can be effectively used to remove false alarms. Experiments are conducted on Remote Sensing Object Detection (RSOD) dataset to compare the proposed method with other advanced methods. The results show that the proposed method can quickly and accurately detect aircraft targets in RSIs and achieve a better performance.

## 1. Introduction

**Figure 1:**
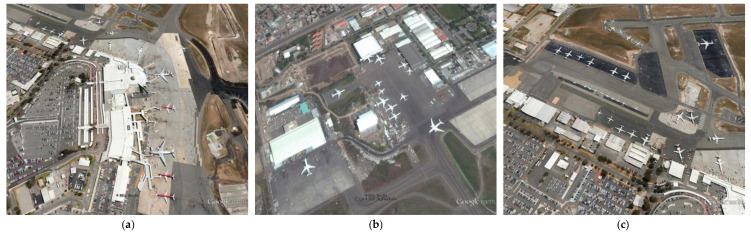
Some aircraft examples in RSIs. (**a**) Complex geo-graphical environmental background; (**b**) aircraft targets in different poses; (**c**) aircraft targets in various sizes.

With the rapid development of image sensing technique [1] and aerospace technology, the acquisition of RSIs has become more convenient. RSIs contain a large amount of useful information, so it is particularly important to fully extract and utilize the information. Owing to the important applications in dynamic airport surveillance and military reconnaissance, aircraft detection in RSIs has attracted much attention [2]. Not only that, for civilian use, effective detection of aircraft targets can improve the utilization of airports while providing guidance on parking areas for aircraft to be landed. Unlike the common natural images, target detection in RSIs has the following specificities: more complex geographical environmental information, variations of target poses and sizes. As shown in Figure 1, all these factors contribute to the degradation of detection algorithm performance.

Recently, various methods have been developed for aircraft detection in RSIs. Those methods can be roughly divided into three categories: attribute-based methods, traditional learning methods, and deep learning methods [3]. As for attribute-based methods, the detection is based on the characteristics of the target. For instance, Liu et al. [4] proposed a template matching aircraft detection method, which used the common feature of aircraft’s cross structure to create a generic template for matching. Due to the wide variations between different aircraft, template matching methods are not accurate. Wang et al. [5] proposed an improved active contour model for RSIs segmentation. Based on convex packets and corner points, the aircraft target was segmented into pieces to be identified. However, this method requires a high level of image segmentation, and it is difficult to achieve effective and accurate segmentation in complex backgrounds.

The traditional learning methods are mostly based on Viola-Jones (VJ) Object Detection Framework [6]. These methods treat target detection as a classification problem. The input to the classifier is a set of target candidate regions with corresponding feature representations, and the output is the corresponding predicted label, namely whether it contains the target object. The whole framework solves the dual problems including discriminating the presence of a target and predicting the location. Research based on this approach has basically focused on two parts: how to generate high-quality target candidate regions and how to design a robust, descriptive feature that can be easily classified. For example, Li et al. [7] proposed a candidate region method by combining a visual saliency algorithm and a spatial competition algorithm and used the directional chamfer matching method to make symmetry detection of potential targets. Similarly, He et al. [8] extracted ship candidate regions in RSIs by segmenting RSIs using visual saliency analysis. While the background of the land is more complex than the sea. Therefore, the region proposal methods for aircraft detection under complex background using visual saliency analysis are not suitable. Liu et al. [9] utilized the Harris corner point detection method in RISs to get potential aircraft regions, and the CNN model was applied to extract features and classify them. But the situation that no aircraft target in RSIs was not considered. If there is no aircraft target in RSIs, the corner points still can be detected, which results in producing a large number of useless candidate regions and consuming a lot of time to classify. For the feature part, Zhao et al. [10] presented an aircraft detection framework based on aggregated channel features, which combined the color channel, normalized gradient channel, and histogram of oriented gradient channel. But it ignored the impact of rotation. Zhang et al. [11] focused on the problem of target rotation and proposed a rotation-invariant parts-based model. Yet the model needs to rotate targets to a fixed principal direction to achieve orientation alignment. This direction normalization method relies on the availability and robustness of the primary direction, which limits the application of the method to arbitrary directions.

Due to the increase in hardware computing ability and the easy access to big data, deep learning methods have achieved great success in natural images. Many studies have also introduced deep learning methods to RSIs analysis. For instance, Ding et al. [12] adopted measures to strengthen the capability of basic VGG16-Net to achieve an improved performance. Wu et al. [13] used the Edge Boxes algorithm to generate region proposals and used the CNN model to extract features and classify them. Wu et al. [14] enhanced the detection effect by adding improved self-calibrated convolution and dilated convolution into the Mask R-CNN framework. Luo et al. [15] proposed the Involution Enhanced Path Aggregation (IEPA) module and Effective Residual Shuffle Attention (ERSA) module, which were systematically integrated into the YOLOv5 base network to improve the aircraft detection accuracy.

With the increasing research on the deep network, some advanced relevant works are proposed in the field of remote sensing. For instance, graph convolutional networks (GCNs) [16] are suitable for multi-label classification, which focus on the relationship between different labels and are more effective in constructing the models of label relevance. Therefore, Hong et al. [17] made improvement on the traditional GCN and developed a new minibatch GCN. The proposed GCN can be trained in minibatch fashion and infer the out-of-sample data without retraining networks. Recently, there has been another well-performing deep network named Transformer [18]. The Transformer network was originally proposed in the field of Natural Language Processing (NLP). Transformer has also achieved good results in various remote sensing tasks. For example, Chen et al. [19] applied the transformer encoder to the modern change detection in RS. While SpectralFormer [20] rethinks hyperspectral image classification from a sequential perspective with transformers. And a highly flexible backbone network was proposed, which provided new insight into the hyperspectral image classification.

Though there are many advantages, deep learning methods require too much labeled data and a long time to complete the training process. Moreover, the implementation of these algorithms requires the support of GPUs and parallel computing. For small platforms such as UAVs, the use of GPUs will increase the carrying burden, power consumption, and economic costs [21]. Therefore, algorithms based on traditional learning are still relevant.

**Figure 2:**
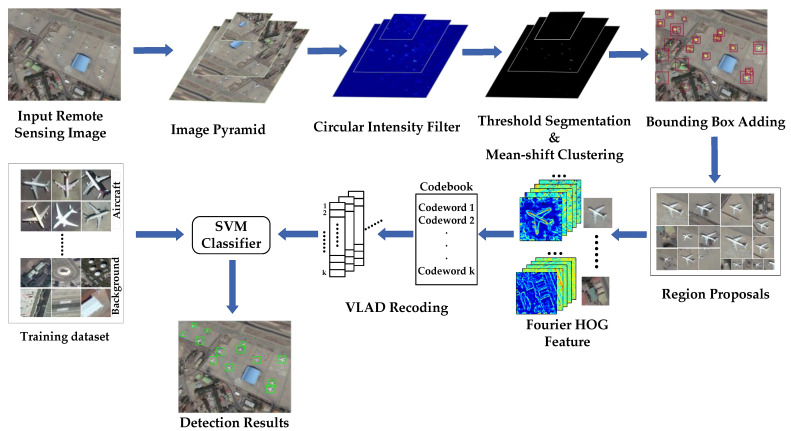
The flowchart of the proposed aircraft detection scheme.

To solve the above problems, a new aircraft target detection scheme is proposed in this paper. The flowchart of the proposed method is shown in Figure 2. The scheme is divided into two parts: region proposal and aircraft target identification. To get multi-scale response magnitude maps, we first construct a circular intensity filter to do convolution with multi-scale RSIs. Then the threshold segmentation and mean-shift clustering algorithms are introduced to get the center point of the target. After adding bounding boxes, the candidate regions are proposed. The proposed region proposal method can quickly locate suspicious targets, and only generate a small number of candidate regions. In the aircraft identification stage, the rotation-invariant HOG descriptor using Fourier analysis in polar coordinates is recoded by Vector of Locally Aggregated Descriptors (VLAD). The new features can be used to classify and identify targets and false alarms quickly. The proposed detection framework achieved a good detection accuracy of aircraft targets in complex scenes and overcomes the problems caused by target rotation. In general, our overall detection method can achieve good experimental results.

The remainder of this paper is organized as follows: Section 2 describes the method for extracting candidate regions in detail, and Section 3 presents the specific steps for improving the Fourier HOG feature. In Section 4, the key parameters determination and performance comparison of the method are presented. In Section 5, the experimental results are discussed. Section 6 concludes the paper and briefly discusses the future direction of the work.

## 2. Aircraft Target Center Determination Based on Circular Intensity Filtering

In this section, we propose a fast convolution method based on circular intensity signal filter to obtain the center-response magnitude maps based on the structural characteristics of the aircraft targets. The threshold segmentation method is applied to operate on the response magnitude maps to get binary images containing the central regions of the aircraft. Then the center points of the target can be determined by clustering algorithm. Candidate regions can be generated by adding specific bounding boxes. In order to get the potential locations of the aircraft with different sizes, we build three-layers image pyramids for the original RSIs and operate on each layer of the pyramid.

### 2.1. Circular Intensity Filter

Due to the dynamics of atmospheric flight, the shape of most aircraft is fixed and can be simply broken down into a nose, a fuselage, a tail, and two wings according to the top view of the aircraft target. The aircraft structure is a cross structure, and the intersection of the fuselage and wings is the center of the aircraft.

**Figure 3:**
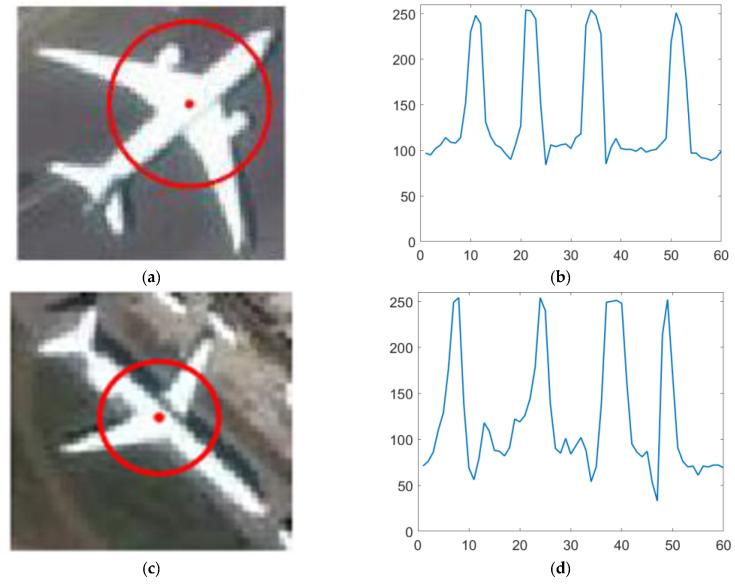
The aircraft and corresponding circular intensity signal waveform charts. (**a**,**c**) Aircraft in RSIs; (**b**,**d**) circular intensity signal waveform charts centered at aircraft’s central point.

If taking the aircraft center as the center of a circle, choose a diameter greater than the width of the fuselage but less than the length of the wingspan to make a circumference. The grayscale value of the image is obtained counterclockwise with this circumference, and it can be found that all the aircraft targets have similar waveforms, as shown in Figure 3. The aircraft target has a stable circular intensity waveform. Each waveform has a phase shift due to different aircraft orientations, but all show a trend of bright-dark-bright-dark-bright-dark-bright-dark.

Let $f_{n}(n=1,2,\ldots ,N-1)$ denotes the gray value of the pixels on the circumference centered at (x,y) and its radius is r. Then do a discrete Fourier transform on this signal and obtain:

(1) $$F=\sum_{n=0}^{N-1}f_{n}e^{-j(\frac{2\pi }{N})kn}$$

The magnitude of *F* is shown as follows:

(2) $${\left|F\right|}^{2}={\left(\sum_{n=0}^{N-1}f_{n}\cos(\frac{2\pi }{N}kn)\right)}^{2}+{\left(\sum_{n=0}^{N-1}f_{n}\sin(\frac{2\pi }{N}kn)\right)}^{2}$$

As is shown in Figure 3, the gray value curve of the circumference centered at the aircraft’s central point has four peaks and four valleys, which is similar to the 4-period sine and cosine curves. So, if the four cycles of sine and cosine functions are selected in Formulas (1) and (2) (that is, k=4), the magnitude value of the circumferential frequency filter in the center of the aircraft is large [22]. Meanwhile, the magnitude avoids the input signal phase interference, and the result is rotation invariant.

If making the circumferential sampling over every pixel in the image, the computational process is complex. To simplify this process, we construct an image convolution template, which is used to achieve the realization of Formula (2). The image convolution kernel can be designed as follows:

(3) $$U=P_{}(r)e^{jk\varphi }(k=4)$$

**Figure 4:**
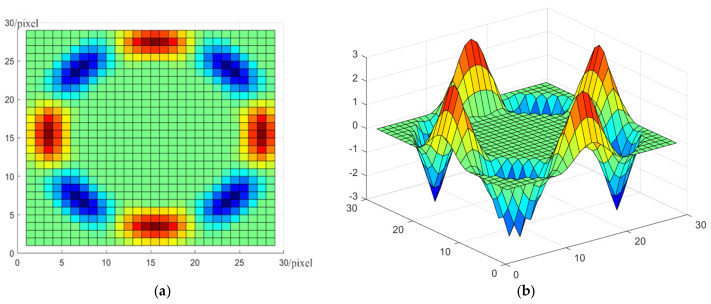
The real part of the constructed convolution kernel. (**a**) The planar schematic; (**b**) the three-dimensional schematics.

The displayed formula consists of a radial function P(r) and a Fourier basis $e^{jk\varphi }(k=4)$. The radial function implements sampling for circumferential pixels in the template. The 4th order circular harmonic function can be decomposed into real and imaginary parts, which are used for correlation convolution operation with the circular intensity signal. The planar and three-dimensional schematics of the real part of the convolutional template (r=13 pixels) are shown in Figure 4.

### 2.2. Centroid Clustering

**Figure 5:**
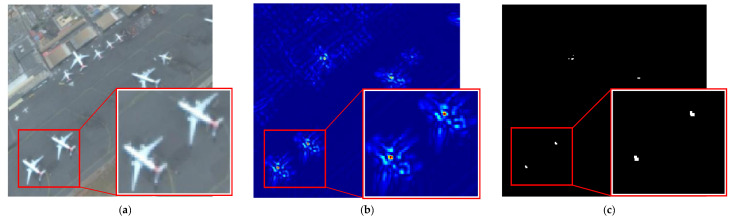
The Original RSI and the process of target center determination. (**a**) The original RSI with the resolution of 1121 × 957 pixels; (**b**) the corresponding center-response magnitude map generated by a convolution kernel (*r* = 20 pixels); (**c**) the binary image with the center of targets obtained by threshold segmentation.

After the convolution operation on the RSIs, the response magnitude maps can be obtained. The response is the correlation of the circumferential gray value signal centered on a pixel and the circumferential gray value approximate signal of the aircraft target. As shown in Figure 5b, the response on the pixel of aircraft’s center is obviously larger than any other pixels. Then threshold segmentation is used to get a binary image containing the center points of the aircraft target. The specific threshold value is determined in the experimental Section 5. As shown in Figure 5c, in the obtained binary image, a large number of speckles in the blob of the center cross structure area are retained. Those points belonging to the same aircraft can be clustered into a group, and the cluster center corresponds to the potential location of the target. Therefore, a clustering algorithm should be used to determine the aircraft targets’ centroid points.

Based on this fact, we introduce the mean-shift algorithm to cluster the points of the central connected region to generate the potential regions of the target. Compared with other clustering methods, the mean-shift algorithm does not require the specification of the number of clusters. The mean-shift algorithm was first proposed by Fukunaga and Hostetler [23], and it is widely used in the fields of data clustering, image classification [24], image segmentation [25], target tracking [26], etc.

In this clustering process, the mean-shift clustering algorithm randomly selects a point as the initial center, and then iteratively finds the probability density maximum point along the direction of the increasing density gradient. The center of mass $m_{h}(x)$ of all points in search radius r is obtained as follows:

(4) $$m_{h}(x)=\frac{\sum_{i=1}^{n}G({\left\|\frac{x_{i}-x}{h}\right\|}^{2})x_{i}}{\sum_{i=1}^{n}G({\left\|\frac{x_{i}-x}{h}\right\|}^{2})},$$

where h denotes the bandwidth, $G({\left\|\frac{x_{i}-x}{h}\right\|}^{2})$ denotes the kernel function.

The mean-shift vector $M_{h}(x)$, denoting the difference between the center of mass $m_{h}(x)$ and the center point x, is calculated by:

(5) $$M_{h}(x)=m_{h}(x)-x$$

If $\left\|M_{h}(x)\right\|$ does not change, the drift process will stop and the mass center is the cluster center. Otherwise, the process is repeated with the mass center as a new center point until convergence.

### 2.3. Multi-Scale RSIs Pyramid

**Figure 6:**
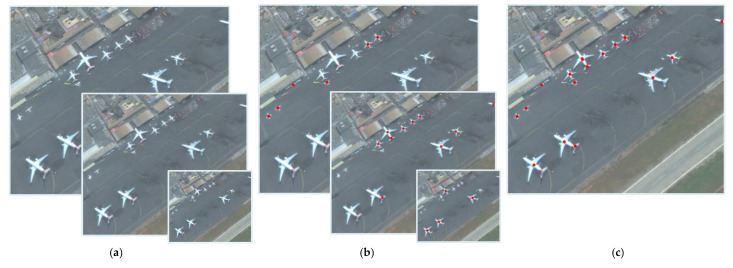
Multi-scale image pyramid and the results of aircraft target center determination. (**a**) RS image pyramid; (**b**) center determination on image pyramid; (**c**) center point aggregation.

There are significant differences in size between aircraft targets even in the same RSI. It is difficult to localize all targets with varying scales in the image for a single circumferential intensity convolution kernel. As shown in Figure 6b, only the small-scale aircraft centers are positioned in the bottom level if using a convolution kernel whose radius is 5 pixels. This will result in low detection recall. Here, we introduce image pyramids to perform multiscale processing on the original RSIs and perform convolution operations on each of the layers of the pyramid. Small targets can be localized in large-scale images. The overall shape of the large target is retained in small-scale images, so they can be localized in small-scale images. Finally, the detected points in the image pyramid can be aggregated to accurately locate multiple targets in RSIs.

## 3. Rotation-Invariant Feature Based on Fourier HOG Feature and VLAD

After obtaining the target center points by the method described above, the candidate regions can be obtained by framing operation. The size of bounding boxes is determined in the experimental part of Section 4. The region proposals can be divided into real aircraft targets and false alarms. The target identification stage aims to distinguish targets from false alarms finely by classifying the features extracted from candidate regions.

In general, the feature extraction operation is to encode the discriminatory information in candidate regions. The features are then fed into a trained classifier for classification, which discriminates between actual targets and false alarms. The rotation of the aircraft can also bring significant differences in the feature representation. The common feature description methods perform poorly, such as the features based on the targets’ color, shape, textures, etc. Therefore, Liu et al. [27] proposed a Fourier HOG descriptor and mathematically proved its rotational invariance. The Fourier HOG feature has been applied to the detection tasks with the rotational interference in the fields of font recognition, remote sensing images, and microscopic imaging [27]. Dong et al. [28] and Yan [6] applied the original Fourier HOG feature to RSIs for ship and aircraft detection, respectively. They all achieved good detection results. Furthermore, Wu et al. [29,30] creatively treated the multidimensional features generated by Fourier HOG as different frequency channel features. The features were effectively integrated with the traditional aggregate channel features (ACF) and the fast pyramid generative model (FPGM). Excellent results were achieved with the help of boosting learning. In this paper, the idea of VLAD sparse representation is introduced to improve the original Fourier HOG from another simple perspective.

This original Fourier HOG descriptor is a pixel-wise feature extraction method of candidate regions, which generates a high dimensionality of features. This can cause problems in the classifying process and may also have the risk of causing memory overflow. We introduce the VLAD encoding method to improve it, which not only reduces the dimensionality of the features effectively but also transforms the original features into higher-level features with statistical properties. The efficiency and accuracy of the classification process are improved.

### 3.1. Fourier HOG

Histogram of oriented gradients (HOG) [31] has proven to be one of the best feature description methods. It has been widely used in the image description field. Fourier HOG method treats histogram of oriented gradients as a continuous signal defined on the angle of 2π and uses the Fourier basis to represent them. This constructs the HOG descriptor with rotational invariance.

The HOG feature is a merged grouping of pixel gradients in an image based on orientation angles, producing a histogram of gradients in discrete directions. The histogram undergoes complex changes as the image rotates. While Fourier HOG feature uses a continuous representation in the gradient direction by creating an orientation distribution function h on each pixel. In the Cartesian coordinates, the gradient d of the pixel (x,y) in an image can be separated as the horizontal component $d_{x}$ and the vertical component $d_{y}$. Let ‖d‖ and Φ(d) be the magnitude and the phase of a complex number $d=d_{x}+jd_{y}$, and the phase can be any value in [0,2π). The distribution function h can be expressed by ‖d‖ and Φ(d) [27]:

(6) h(φ)=‖d‖δ(φ−Φ(d))

The distribution function h(φ) is a period of orientation with a period of 2π, so it can be formulated by using its Fourier series coefficients:

(7) $$h(\varphi )=\sum_{m=-\infty }^{\infty }a_{m}e^{jm\varphi }$$

where $a_{m}=\frac{1}{2\pi }\int_{0}^{2\pi }h(\varphi )e^{-jm\varphi }d\varphi =\left\|d\right\|e^{-jm\Phi (d)}(m\in {\mathbb{Z}}_{0,M})$.

Limiting the value of the maximum frequency order |m| is equivalent to low-pass filtering in the frequency domain, which provides a “soft binning” smoothing effect.

**Figure 7:**
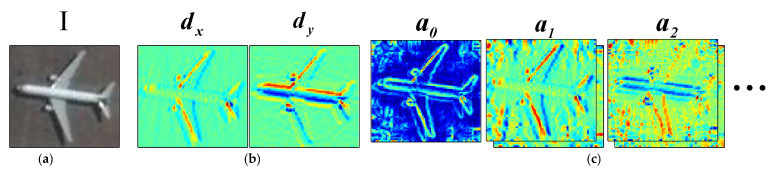
Illustration of the expansion of gradient images to Fourier coefficient images. (**a**) The input image; (**b**) the gradient image; (**c**) the complex Fourier coefficient images.

Thus, a series of complex coefficient images can be generated based on the gradient images. An example of this expansion is shown in Figure 7.

A series of basis functions are constructed to do convolution with the gradient Fourier coefficient map and get the convolution result $U_{i,k}*a_{m}$. The basis function is a combination of a triangular kernel with isotropic and circular harmonic filters, which can be indicated as:

(8) $$U_{i,k}(r,\varphi )=\Lambda (r-r_{i},\sigma )e^{jk\varphi }$$

where Λ is a triangular function of width 2σ defined as $\Lambda (x,\sigma )=\max(\frac{\sigma -\left|x\right|}{\sigma },0)$.

Based on the derivation of the literature [27], a rotation-invariant feature can be constructed consisting of the following three components. If k−m=0, the rotation order of the convolution results is 0, so the result is rotation-invariant. If k−m≠0, the amplitude of the convolution result is rotation invariant. The third component of the rotation-invariant feature is obtained by coupling two different convolutional results, and this satisfies the formulation:

(9) $$\bar{\left(U_{i_{1},k_{1}}*a_{m_{1}}\right)}(U_{i_{2},k_{2}}*a_{m_{2}}),\forall k_{1}-m_{1}=k_{2}-m_{2}$$

### 3.2. VLAD Representation

The aforementioned Fourier HOG feature creates an orientation distribution function on each pixel and is obtained by convolution in full image. The final generated features are pixel-wise, and with a lot of non-discriminative and redundant information.

The VLAD representation is a popular image coding method, which can aggregate descriptors into a fixed-size dimension based on a local aggregation principle in feature space. The representation method is first proposed by Jegou [32], and it is mainly used in the field of image retrieval. It has advantages over the widely used Bag of Words (BoW) [33] method in terms of retrieval accuracy and reduced computational effort compared to the Fisher Vector (FV) [34] method. Therefore, we choose the VLAD method to recode Fourier HOG features.

The idea of VLAD is similar to BoW. The extracted local features are first clustered into several groups. The classical BoW approach is represented by a histogram, and the value of each bin is the number of features belonging to each particular group. While the VLAD method is a vector of residuals sum between the center of mass of each group and the local features belonging to that group.

**Figure 8:**
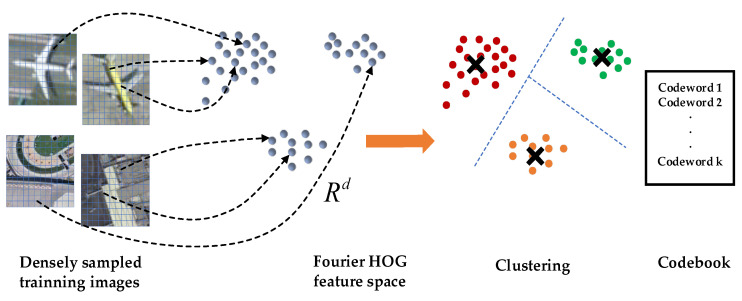
Illustration of the codebook generation in the VLAD representation.

The VLAD representation of the Fourier HOG feature consists of the following steps. As shown in Figure 8, a training data set with a large number of positive and negative samples is first given, and dense extraction of Fourier HOG features for each image patch is carried out. The Fourier HOG feature of each pixel is indicated as $x_{i}(i=1,2,\cdot \cdot \cdot ,N)$. To construct a codebook, the K-Means algorithm is applied to cluster all features into k clustering centers. $c_{m}(m=1,2,\ldots ,k)$ is the center of clusters. Similar to the representation of BOW, each local descriptor $x_{i}$ is assigned to its nearest codeword, and the quantified indexes are then obtained:

(10) $$\mathrm{NN}(x_{i})=\arg\min_{m}\left\|x_{i}-c_{m}\right\|$$

Then accumulating the difference between the center of each cluster and its contained descriptors subsets to obtain the vector:

(11) $$u_{m}=\sum_{i:\mathrm{NN}(x_{i})=m}x_{i}-c_{m}$$

The final feature descriptor $U(u_{1},u_{2},\ldots ,u_{m})$ is obtained by connecting all vectors $u_{m}$.

The images in the test set are also extracted to get their Fourier HOG features and then represented in the feature space using the codebook formed by the training set.

## 4. Experiments

In this section, experiments are conducted to validate the performance of the proposed aircraft target detection method. First, we give a brief description of the dataset used in the experiments and then identify some key parameters of the region proposal method. Finally, the effectiveness of the overall detection framework is verified by comparing it with other commonly-used methods. All the experiments were implemented on the platform of Intel Core i7-10700F @2.90GHZ CPU (Santa Clara, CA, USA)and 32 GB RAM.

### 4.1. Dataset and Evaluation Criteria

The optical RSIs used in this paper are derived from the RSOD dataset, which contains 446 remote sensing images with a total of 4993 aircraft targets. The sizes of the images are 1072 × 975 and 1116 × 659. Those images are from Google Earth and Tianditu, whose spatial resolutions range from 0.5 m to 2 m. Please refer to [35,36] for more details.

In our experiments, 60% of the image samples are assigned as the training set, and the rest are assigned as test sets. In the training set, images are randomly selected to determine the size of the candidate regions, as well as to evaluate the performance of the candidate regions extraction.

To evaluate the performance of the proposed method, the intersection-over-union (IoU) between detection result and ground truth is adopted in this paper. When the IoU is greater than or equal to 0.5, the detection result is considered to be correct. We use the Precision-Recall curve (PR curve) and Average Precision (AP) to assess the performances of the proposed overall aircraft detection framework. The precision and recall are calculated with the following formulas:

(12) $$Recall=\frac{TP}{TP+FN}$$

(13) $$Precision=\frac{TP}{TP+FP}$$

where *TP* denotes the number of true positive aircraft recognition targets, *FP* denotes the number of false positive aircraft recognition targets, *FN* denotes the number of false negative aircraft recognition targets.

Average Precision (AP) is a measure that combines recall and precision for ranked detection results. AP computes the average precision value for recall value over 0 to 1. Namely AP is the area under curve (AUC). Let *r* represents recall and *p(r)* represents corresponding precision in the PR curve. Then the AP can be calculated:

(14) $$\mathrm{AP}=\int_{0}^{1}p(r)dr$$

### 4.2. Parameter Settings and Comparison Experiments for Region Proposals

#### 4.2.1. The Size of Bounding Boxes

Based on the description above, the center points of potential aircraft target regions can be obtained by circular intensity filtering and a series of operations. Taking these points as the center points of the bounding boxes, the candidate regions can be obtained by cutting into several image patches with certain specific sizes. The size of bounding boxes has a significant impact on the accuracy of target detection. Because too large size will result in a patch containing too much background interference, and too small size will result in incomplete aircraft structure. To determine the proper size of bounding boxes, we analyze the long side of the target ground truth in the training dataset.

**Figure 9:**
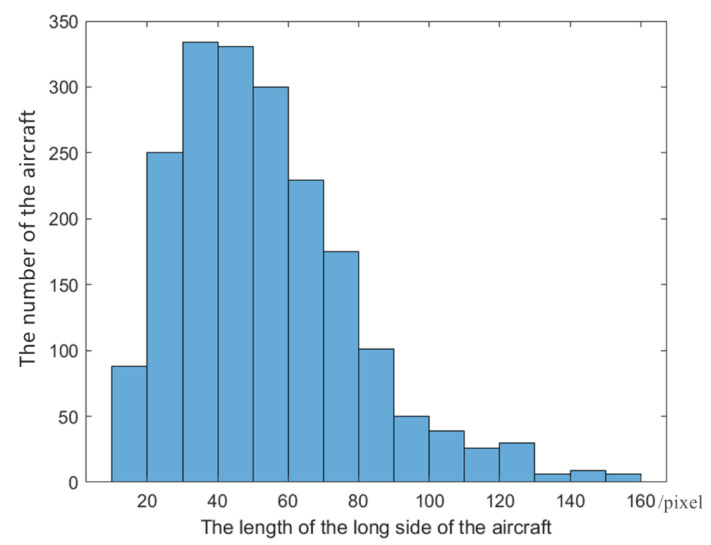
The distribution histogram of the aircraft target long side.

To facilitate feature extraction and classification, aircraft targets are generally discussed in square form [13]. As shown in Figure 9, the length of most target regions lays between 10 pixels and 130 pixels. Therefore, we choose to use multiple sizes to crop the original RSIs to get candidate regions. Since a three-level image pyramid is created, it is possible to specify the number of categories for clustering the long side of the aircraft. K-means method is used here and set the number of categories K to 3 for clustering. Three clustering centers of the length can be obtained: 34.8, 76.21, and 119.87. Thus, we can set 40, 80, 120 as the length of three-size bounding boxes, and the candidate regions’ sizes are 40 × 40, 80 × 80, 120 × 120.

#### 4.2.2. Thresholds Determination in the Segmentation

We construct image pyramids of the original RSIs at three different scales, and the convolution kernel combining the radial function and 4th order circular harmonic function. Set the radius of the convolution kernel to 5. The convolution operation is performed on each layer of the image pyramid to get the response magnitude map pyramid. To remove the background interference in the image to get the true aircraft target center, it is necessary to perform threshold segmentation on each scale. An overly high threshold will filter out the true targets that are available, making the recall rate lower. A too low threshold retains a large number of false alarm centroids, giving burden on the later classification stage. The recall of the region proposal stage has a direct influence on the final detection performance. We conduct experiments on the region proposal stage with the different combinations of three thresholds, which is used in three levels RSIs pyramid. The results are shown in Table 1. It is possible to conclude that the combination of t_0_ = 0.3, t_1_ = 0.3, t_2_ = 0.5 is a trade-off result between recall and candidate regions number.

#### 4.2.3. The Comparative Experiments of Region Proposal Method

The proposed region proposal method based on circular intensity filter is compared with the two most popular region proposal methods including the EdgeBoxes algorithm [37] and the Selective Search (SS) algorithm [38]. For the parameter setting of the compared algorithms, refer to the corresponding references for more details. The result is shown in Table 2. From Table 2, it is easy to conclude that the proposed region proposal method gets the best performance in terms of the number of candidate regions and recall. The time cost of our method is equal to the EdgeBoxes algorithm and far superior to the SS algorithm. The reasons are listed as follows. The EdgeBoxes algorithm focuses on the contour information of the target object. Due to the imaging problems such as uneven illumination, which causes the targets’ edges unclear and contour incomplete, the recall of the EdgeBoxes algorithm has worse performance. The sliding windows method is used in the EdgeBoxes algorithm to extract candidate regions. Though it has a faster speed, it can also generate a large number of regions, which is not friendly to the subsequent classification. SS algorithm uses the graph-based method to segment the RSI image to get different candidate regions. This segmentation algorithm produces good results, but its complexity causes the algorithm to be time-consuming. Compared with the two algorithms, our proposed method constructs convolution kernels for the significant structural properties of the aircraft target, which is robust to complex backgrounds and disturbances in RSIs and produces only a small number of high-quality candidate regions.

### 4.3. Comparison of Overall Detection Performances

**Figure 10:**
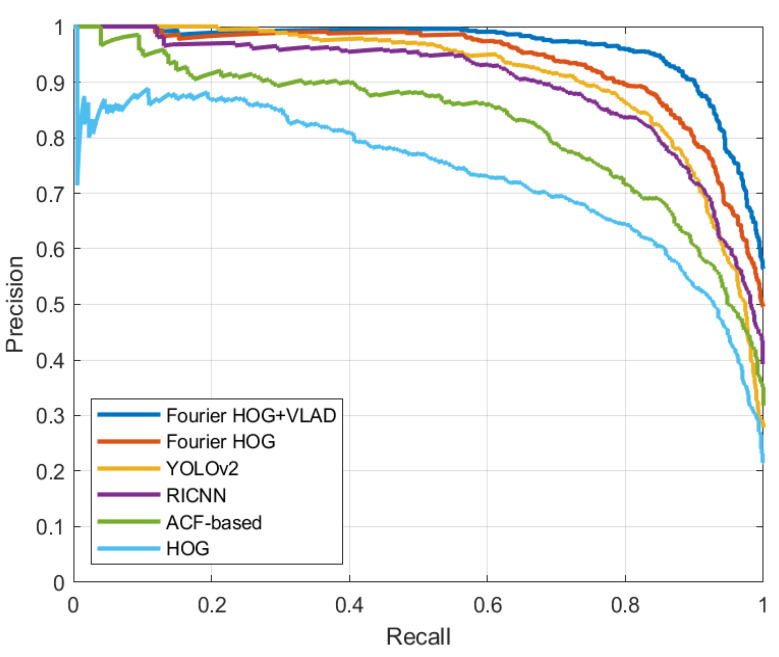
The PR curves of different methods on the RSOD dataset.

To quantitatively evaluate the performance of the overall detection method, we compare several state-of-the-art methods related to our proposed framework, such as ACF-based [10], and two other deep-learning methods. One is RICNN [39] that is based on Convolutional Neural Networks (CNN) model and rotation invariant analysis, and the other is YOLOv2 [40]. To verify the effect of the Fourier HOG-VLAD feature, the methods based on HOG and Fourier HOG feature are conducted to make a comparison. For the sake of fairness, our proposed region proposal method is used instead of the sliding windows method used previously. For the parameter settings of these algorithms, please refer to the original cited literature. The PR curves of the different methods on the RSOD dataset are shown in Figure 10, and Table 3 correspondingly lists the quantitative results in terms of APs and mean running times.

According to Figure 10 and Table 3, we can observe that the HOG and ACF-based methods get the worst performance. Because both of these methods ignore the rotational behavior of the aircraft target. When the image rotates, the gradient feature sampled in discretized grid has complex changes. Deep learning networks can extract deep semantic information, which is more helpful to locate the target accurately. But RICNN uses the SS algorithm to extract candidate regions, which costs lots of calculation time. The network of YOLOv2 has been carefully designed and it performs best in runtime. But its network is not robust to tiny, arbitrarily oriented objects. Although we use data enhancement operations such as random flip, photometric, and geometric distortion in our experiments, the relative training data are still far from sufficient. Due to the dual effect of the high-quality region proposal method and the strong representational ability of the rotation-invariant feature, the detection method based on the original Fourier HOG feature outperforms the two deep learning methods. But due to the high dimensionality of the Fourier HOG feature, the method based on the Fourier HOG feature gets the longest running time. The proposed framework introduces VLAD to represent the Fourier HOG feature, which effectively reduces the dimensionality of the features and improves the feature presentation ability by transforming them into higher-level ones. The improvement obviously reduces the overall detection process time. For the AP evaluation metric, the proposed method outperforms all other methods. In addition, the proposed approach does not rely on large amounts of training data and dedicated computing platforms such as GPUs.

**Figure 11:**
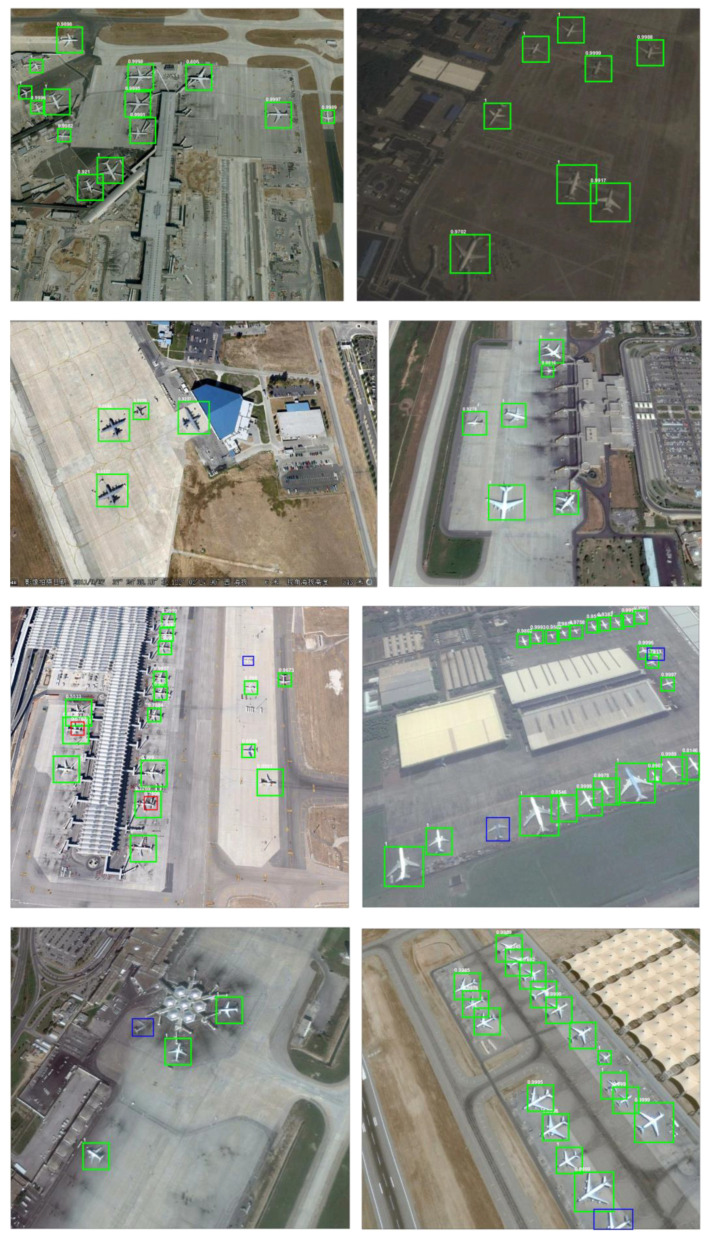
Some visual detection results of the proposed method in the RSOD dataset.

The visualization of detection results in the testing set is shown in Figure 11, where the green border indicates the correct detection targets, the red border indicates the wrong detection results, and the blue border indicates the missed detection aircraft. In most cases, the proposed method in this paper can accurately determine the position of the aircraft target. Even in the case of insufficient light in the Figure 11 below, the method can still achieve a good detection result. However, there are also target misdetections and missed detections. The results of the failure are analyzed as follows. The situations with too complex background, such as the concourse of the terminal building, the shadow of the aircraft tail may lead to the target misdetections. On the other hand, due to the unevenness of light and aircraft coating, the aircraft fuselage color is close to the ground color, which will also result in some missed detections. In Figure 11, problems such as cloud obscuration and RSI clipping can also result in the inability to determine the center of the aircraft, which can lead to missed detection.

## 5. Discussion

Recently, deep learning methods have achieved great success in various tasks in remote sensing. It is inescapable that deep learning methods requires large amount of labeled training data and the support of specialized processing platforms like GPUs. Based on a small sample dataset, we propose a novel aircraft detection method on the basis of traditional machine learning VJ architecture, which provides a simple and easy idea to implement.

In the region proposal stage, we introduce the idea of correlation filtering to construct a circular intensity filter to do fast convolution with the whole RSI. The convolutional response at the center of the aircraft is much higher than the other positions. So that the candidate regions can be extracted quickly. According to Table 2, compared with the two popular region proposal methods (SS algorithm and EdgeBoxes algorithm), our proposed method has a huge advantage in the number of candidate regions and the recall rate. The two methods generate large quantities of candidate regions without categories. In terms of time cost, the proposed method is almost comparable to the EdgeBoxes algorithm.

In the target identification stage, namely the fine screening stage, we apply the sparse representation method VLAD to improve the rotation-invariant Fourier HOG feature. According to the Figure 10 and Table 3, the proposed method improves the detection performance and reduces the detection time cost compared to the original feature. However, the time cost is still not the optimal.

We have calculated the computational complexity of the method. Given an RSI image with M pixels, the complexity of the filtering is *O*(NM), where N is the pixel number of the circular intensity convolutional template. The complexities of threshold segmentation and mean-shift clustering are *O*(M), *O*(Tn^2^), respectively, where T is the number of iterations, n is the number of pixels of 1 in binary image. The complexity of the region proposal stage is *O*(NM) + *O*(M) + *O*(Tn^2^). Assume that an image patch generated by region proposal stage has m pixels. The complexity of the Fourier HOG feature extraction is *O*(skmn), where s is the scale factor of the basis function, k is the order of the basis function, and n is the pixel number of the basis function convolutional template. The complexities of VLAD and linear SVM (except for the offline learning process) are *O*(2KD(p+1)) [41], *O*(d) [42], respectively, where K is the number of clusters, D is the dimension of the feature, p is the number of the local feature, and d represents the dimension of the input data to linear SVM. So, the complexity of the target identification is *O*(skmn) + *O*(2KD(p + 1)) + *O*(d). In the future, we will focus on hardware acceleration strategies to improve the detection speed.

## 6. Conclusions

Aircraft target detection in RSIs is a challenging problem due to the complex background and the variation of target size and direction. In this paper, we propose a novel aircraft target detection framework in which a region proposal method based on the circular intensity filter is constructed to locate potential multi-scale aircraft targets in RSIs. Moreover, we use the VLAD method to represent the rotation-invariant Fourier HOG feature, which has the lower dimensionality and the stronger description ability insusceptible of the target’s rotational behavior. Compared with other popular methods, the proposed method produces fewer high-quality candidate regions, while the overall detection method has better performance and is more robust to aircraft deformation. In future work, we will focus on small target detection, and improve the method to reduce the impact of uneven illumination and occlusion.

## Figures and Tables

**Table 1:** The results of region proposal method under different parameter t_0_, t_1_, t_2_ settings.

Combinations of thresholds	t_0_ = 0.2	t_0_ = 0.3	**t_0_ = 0.3**	t_0_ = 0.35	t_0_ = 0.4	t_0_ = 0.4
	t_1_ = 0.2	t_1_ = 0.3	**t** **_1_ = 0.3**	t_1_ = 0.35	t_1_ = 0.4	t_1_ = 0.4
	t_2_ = 0.4	t_2_ = 0.4	**t_2_ = 0.5**	t_2_ = 0.5	t_2_ = 0.4	t_2_ = 0.5
Number of candidate regions/Per image	235	181	**163**	127	78	73
Recall	0.984	0.976	**0.976**	0.935	0.911	0.91

**Table 2:** The results of the three different region proposal methods.

Method	Number of Candidate Regions per Image	Recall	Time (s) per Image
EdgeBoxes	6109	0.928	**0.426**
Selective Search	3892	0.941	11.446
Proposed	**155**	**0.953**	0.513

**Table 3:** The performance comparisons of different methods on the RSOD dataset.

Method	HOG	ACF-Based	RICNN	YOLOv2	Fourier HOG	Proposed Method
AP	0.697	0.808	0.874	0.881	0.905	**0.934**
Mean Time (s) per Image	0.72	2.23	8.84	**0.15**	2.37	1.31

## Data Availability

The dataset presented in this study are available through: https://github.com/RSIA-LIESMARS-WHU/RSOD-Dataset- (accessed on 28 November 2021).
